# On the feasibility of the sonochemical preparation of hierarchical faujasite-type zeolites as an alternative to ultrasonic‑assisted alkaline treatment

**DOI:** 10.1016/j.ultsonch.2026.108046

**Published:** 2026-09-08

**Authors:** Łukasz Kuterasiński, Małgorzata Ruggiero-Mikołajczyk, Małgorzata Zimowska, Mariusz Gackowski, Grzegorz Kurowski, Przemysław Jakub Jodłowski

**Affiliations:** a Jerzy Haber Institute of Catalysis and Surface Chemistry, Polish Academy of Sciences, ul. Niezapominajek 8, 30-239 Kraków, Poland; b Faculty of Chemical Engineering and Technology, Cracow University of Technology, Warszawska 24, 31-155 Kraków, Poland

**Keywords:** Sonication, Faujasite, Crystallinity, Hierarchization, Fragmentation

## Abstract

Using sonochemistry to synthesize hierarchical faujasite-type zeolites could offer a promising alternative to traditional alkaline-solution manufacturing. This approach could eliminate the need for costly and often hazardous reagents, thereby lowering production expenses and supporting green chemistry initiatives. Unlike conventional zeolite hierarchization, this innovative method could replace alkaline desilication of microporous zeolites entirely with ultrasonic pretreatment of the gel, followed by hydrothermal crystallization of the zeolite product. Selecting faujasite from over 260 possible zeolite structures highlights its broad utility in the chemical industry. The synthesized systems were analyzed for crystallinity, structure, Si/Al ratio, chemical states of silicon and aluminum, morphology, and acidity. Results showed that increasing ultrasound exposure time led to smaller crystallites and reduced microporosity, resembling effects obtained via standard alkaline desilication of parent microporous zeolites. Hence, it could be concluded that the sonicated zeolites were hierarchical materials at first sight. Nevertheless, significant drops in microporosity, accompanied by distinct fragmentation and a relatively low drop in Si/Al ratios, suggested existing interparticle voids rather than intracrystalline mesopores and macropores. Therefore, the sonicated materials reported in this work can be described as seemingly hierarchical faujasites. Furthermore, the sonicated faujasites exhibited high sensitivity to further modification, prompting further optimization studies, including zeolite structures with higher starting Si/Al ratios.

## 1 Introduction

Zeolites are crystalline microporous aluminosilicates that, aside from silicon, aluminum, and oxygen, can contain protons and metal cations from both the first and second groups of the periodic table, as well as transition metals [1]. The International Zeolite Association (IZA) recognizes over 260 distinct zeolitic structures, each exhibiting unique physical and chemical characteristics [2]. Their active sites make them valuable in many industrial catalytic processes [3], [4].

These aluminosilicates offer several advantages: zeolites are straightforward to synthesize, easily modified, and their production is fully reproducible. Their broad appeal in industry stems from their high thermal resistance and shape selectivity [5]. However,

a significant drawback for catalytic applications is the slow movement of reactants within their narrow pores and chambers, leading to rapid catalyst deactivation [6], [7], [8]. Increasing pore sizes to create hierarchical structures—comprising micropores, mesopores, and occasionally macropores—can address this issue. Such modifications use alkaline solutions, surfactants, carbon particles, or various organic compounds. Chemically, hierarchical materials are generated by removing silicon (and sometimes aluminum), creating defects in their structure [9], [10], [11], [12], [13], [14], [15], [16], [17], [18], [19], [20], [21], [22], [23], [24], [25], [26].

The literature suggests that ultrasonic irradiation is an effective method for producing zeolite-based hierarchical materials due to its distinctive reaction pathways, which broaden material development possibilities. The ultrasonic technique relies on cavitation in liquids, generating localized “hot spots” with extremely high temperatures and pressures. Under these conditions, particles move rapidly, collide, and form radicals, triggering further sonochemical reactions. Sonication not only shortens synthesis times and reduces energy consumption but also minimizes the need for costly or hazardous reagents, lowering overall production costs [27], [28], [29], [30], [31], [32].

Sonochemistry has shown positive results in preparing and modifying LTA, MCM-22, CHA, FAU, MFI, and BEA zeolitic structures [33], [34], [35], [36], [37], [38], [39]. Typically, ultrasonically synthesized zeolites feature improved crystallinity and smaller crystals achieved within shorter periods. For metal-containing zeolites, sonication has been used to deposit copper, cobalt, or iron species onto FAU, ZSM-5, and BEA types [40], [41], [42], [43]. Ultrasonic preparation resulted in superior distribution of metal compounds compared to conventional methods, with copper and cobalt forming Cu-O-Cu or Co-O-Co dimers, and iron forming Fe_x_O_y_ species [43].

Ultrasonic irradiation has also been applied to create hierarchical zeolite materials. Oruji et al. [44] studied hierarchical NaY zeolite produced via ultrasonic-assisted alkaline treatment and observed increased mesoporosity with longer desilication times, though at the expense of crystallinity. Zhang et al. [45] synthesized mesoporous zeolite Y through initial dealumination followed by ultrasonic-assisted desilication—ultrasound was notably more efficient than traditional approaches. Khoshbin et al. [46], [47] developed mesoporous ZSM-5 using ultrasound and rice husk ash as a silica source, along with carbon nanotubes as

a template. Initial ultrasonic treatments preserved crystallinity and morphology while boosting mesoporosity, but extended exposure led to crystallinity loss and greater surface area.

An ultrasonic-assisted desilication of Al-deficient FAU-type zeolite, described in [48], created acid sites with unprecedented strength (>410 cm^−1^). Subsequent research confirmed that longer desilication times led to reincorporation of silicon during ongoing alkaline treatment [49], demonstrating that both extraction amounts and agent composition can be adjusted to control Si and Al removal.

The choice of zeolite structure strongly influences the effects of alkaline treatment, whether ultrasound is used or not. Faujasite showed much higher silicon leaching (∼40%) compared to ZSM-5 (∼9%), but ZSM-5 had greater aluminum removal (up to 15% vs. 1–3% for FAU). These differences relate to porosity formation; FAU developed more mesoporosity and underwent channel-like hierarchization—resulting in pronounced grain fragmentation—while ZSM-5 showed point-like hierarchization similar to pumice grain formation [48], [50].

This research advanced the field by developing a sonochemical method as a potential way to prepare hierarchical faujasite-type zeolites, which could offer a promising alternative for manufacturing these systems through sonically assisted alkaline desilication. This process could eliminate the need for post-synthesis modification of microporous zeolites, avoiding costly and harmful chemicals and benefiting the environment. The suggested ultrasonic preparation for FAU-type hierarchical zeolites might be achieved by the sonication of the gel followed by hydrothermal crystallization. The sonicated zeolites obtained in this way could be used as active and selective catalysts. In the current research, the tested reaction was the decarbonylation of furfural into furan, because furfural is a key precursor for the production of polymers, resins, adhesives, fuels, coatings, dyes, antibiotics, food additives, solvents, and many other valuable industrial chemicals. Therefore, furfural processing practically affects everyone living in the civilized world.

## 2 Experimental

### 2.1 Preparation of samples

The synthesis of FAU-type zeolite was conducted by introducing colloidal silica (Ludox AS 40%) into sodium aluminate (Riedel-de Haën, ≥98%), which had previously been dissolved in an aqueous NaOH solution (Sigma-Aldrich, ≥98%) under vigorous agitation. The prepared zeolite gel possessed a molar chemical composition, expressed in oxides, of 35Na_2_O:1470H_2_O:19Al_2_O_3_:100SiO_2_. Following preparation, the gel was sealed in a polypropylene beaker and maintained at ambient temperature for 24 h. After aging, the precursor gel underwent ultrasonic treatment for durations of 30, 60, or 90 min. Subsequently, the treated gel was placed into a Teflon-lined stainless-steel autoclave, sealed, and subjected to static conditions at 95 °C for 24 h. Samples were designated as FAU(30), FAU(60), and FAU(90), corresponding to the ultrasonic treatment duration (expressed in minutes). The reference sample, FAU(0), was synthesized via standard hydrothermal methodology.

Ultrasonic pretreatment of each zeolite gel sample was performed with a QSonica Q700 sonicator (Church Hill Rd, Newtown, CT, USA), operating at an effective power of 60 W, acoustic power density of 1.2 W/mL, acoustic power intensity of 47.4 W/cm^2^, and a frequency of 20 kHz. During sonication, the gel and probe assembly were immersed in an ice bath to ensure maintenance of room temperature throughout the procedure.

### 2.2 Physicochemical characterization of samples

Zeolite crystallinity was assessed by X-ray diffraction (XRD) using a PANalytical X’Pert PRO MPD diffractometer (40 kV, 30 mA), equipped with a CuKα generator (λ = 1.5418 Å). Scans were conducted over a 2θ range of 5–50°, with a step size of 0.033°. Powdered zeolite samples were mounted in holders for analysis. The crystallinity of the samples was evaluated by integrating the seven most intense reflections in the XRD patterns, attributed to the (1 1 1), (3 1 1), (3 3 1), (4 4 0), (5 3 3), (6 4 2), and (7 5 1) planes, respectively. Average crystallite sizes were calculated via the Scherrer Eq. (1), utilizing data from HighScore software integrated with the diffractometer.

(1) L=λK/βcosθ

where *λ* is the X-ray wavelength (1.5418 Å); *K* is the dimensionless shape factor (0.9); *β* denotes the full width at half maximum (FWHM); and *θ* represents the Bragg angle (6.2°; 11.9°; 15.6°; 20.3°; 23.5°; 26.9°; and 31.2°). The calculated crystallite sizes automatically included strain analysis, according to the Williamson-Hall model.

The silicon environment in the samples was investigated using solid-state ^29^Si MAS NMR spectroscopy on a Bruker Avance III spectrometer (500 MHz, 11.7 T, 99.4 MHz) at an 8 kHz spinning rate. Measurements employed zirconia rotors with high-power proton decoupling (SPINAL64), 5.8 μs (π/3) pulses, and a repetition time of 20 s. Chemical shifts were referenced to tetramethylsilane (TMS; >99%). Aluminum speciation was analyzed via ^27^Al MAS NMR spectra at a spinning rate of 10 kHz, utilizing a 0.2 μs (π/12) pulse and a recycle delay of 0.1 s. Chemical shifts were referenced against aqueous 1 M aluminum nitrate. Samples were fully hydrated at room temperature in the presence of vapor-saturated magnesium nitrate before NMR experiments.

Chemical composition, specifically the Si/Al ratio, was determined by ICP-OES elemental analyses using an Optima 2100DV instrument (PerkinElmer).

Porosity assessments were performed using nitrogen adsorption at −196 °C with the Autosorb-1 Quantachrome apparatus. Specific surface area (S_BET_) was determined by the application of the Barrett-Joyner-Halenda (BJH) model (at 0.05 < p/p_o_ < 0.35); the micropore volume (V_micro_) was calculated using the t-plot method (at 0.1 < p/p_o_ < 0.35) with de Boer statistical thickness ranging between 3.8 Å and 5.1 Å, whereas pore size distribution was calculated using Density Functional Theory (DFT) – Monte Carlo Differential Pore Volume Distribution models. Total pore volume was determined at p/p_o_ = 0.994 according to the Gurvich model. The meso- and macropore volumes in the studied samples were calculated as the subtraction result between total pore volumes and micropore volumes. Samples underwent degassing under vacuum at 250 °C overnight before measurement.

Morphological studies were performed using a Nova Nano SEM 300 FEI microscope. Before SEM analysis, samples were dried for 24 h and coated with a thin carbon layer. The microscope was equipped with an Energy Dispersive Spectroscopy system (EDS).

Transmission electron microscopy analysis (TEM) was conducted using a JEOL JEM 2100 HT LaB6 (JEOL USA, Inc., Peabody, MA, USA), with an accelerated voltage of 80 kV and a spot size of 1 nm. Before TEM analyses, the studied samples were sprayed onto Formvar film-coated copper grids.

Quantitative determination of Brønsted and Lewis acidity was achieved through Fourier Transform Infrared Spectroscopy (FT-IR) employing a NICOLET iS10 spectrometer (Thermo Scientific) with an MCT detector. Analyses were conducted in the 4000 cm^−1^ to 650 cm^−1^ wavenumber region at a resolution of 4 cm^−1^ and 128 scans per spectrum. Thin wafer samples (∼70 mg) were activated in vacuum for 1 h at 400 °C, using a temperature ramp of 5 °C/min. Subsequent quantitative analysis involved the sorption of ammonia (Air Products, 99.95%) at 120 °C, monitored by FT-IR. Concentrations of Brønsted and Lewis acid sites were calculated from band intensities attributed to ammonium ions or ammonia bonded to Lewis sites, respectively. The determined extinction coefficients were: 0.12 cm^2^/μmol for the 1450 cm^−1^ band (Brønsted acidity) and 0.026 cm^2^/μmol for the 1620 cm^−1^ band (Lewis acidity) [48]. For the determination of acidic strength, the sorption of ammonia was conducted at 120 °C − 240 °C, with a step of 20 °C. The ammonia sorption experiments were preceded by the recording of FT-IR spectra of the activated samples (at 400 °C) under the same temperature conditions.

The catalytic performance of the prepared samples in furfural decarbonylation to furan was evaluated in a fixed-bed quartz microreactor at atmospheric pressure and 250–350 °C. In each run, 0.5 mL of catalyst (400–500 μm) was loaded onto a quartz wool plug and reduced under pure hydrogen (50 ml/min) at 400 °C for 2 h. The reaction was then carried out using a hydrogen stream containing furfural (0.23 mg/min) at 25 ml/min, with a total flow rate of 50 ml/min and a WHSV of 6000 h^−1^. The outlet gas composition was monitored continuously by gas chromatography using an SRI8610A instrument equipped with an FID detector.

## 3 Results and discussion

### 3.1 Sonication and its mechanism vs. physicochemical properties

**Fig. 1:**
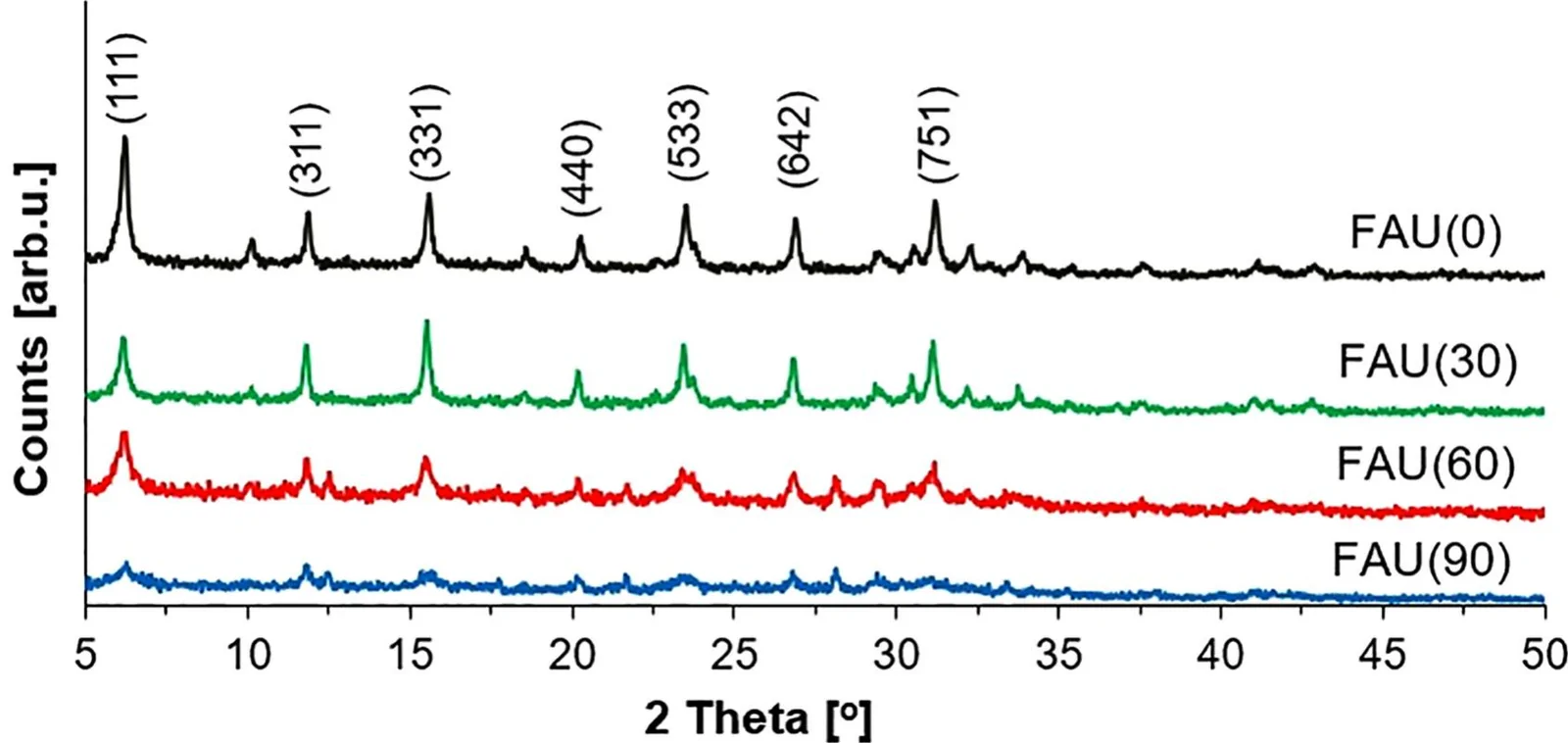
XRD patterns illustrating the influence of the exposure time of zeolite gel to ultrasound on the crystallinity of the prepared zeolite systems.

XRD patterns of zeolite samples prepared through ultrasonic-assisted gel treatment, followed by aging and hydrothermal processes, are illustrated in Fig. 1. Analysis of these XRD data confirmed the presence of a cubic crystalline faujasite phase with space group F (00-038-0238 JCPDS card) [51]. Sonochemical treatment of the aged gel precursor influenced the crystallinity of the resulting zeolite product. The duration of ultrasound exposure was found to be a critical factor; extended ultrasonic processing (from 0 to 90 min) led to a marked decrease in the crystallinity of the zeolite (from 100% for the reference sample to 23%) and a concurrent reduction in average crystallite size (from 544 Å to 177 Å), as detailed in Table 1. Interestingly, the lattice strain analysis indicated that a longer duration of ultrasonic pretreatment of the gel resulted in stronger lattice strain effects (from 0.5% to 2.1%), which corresponded to the loss of crystallinity.

**Table 1:** The impact of the ultrasonic pretreatment duration of zeolite gel on the structure, chemical composition, and relative yield of the prepared zeolite systems.

Sample	Structure			Si/Al			Relative sample yield [%]
	Crystallinity [%]	Crystallite sizes [Å]	Average lattice strain [%]	ICP	EDS	gel	
FAU(0)	100	544	0.5	2.47	1.70	2.65	100
FAU(30)	85	519	0.6	1.86	1.71		96
FAU(60)	51	262	1.0	1.85	1.73		94
FAU(90)	23	177	2.1	1.54	1.80		80

Comparable effects were observed by Safari et al. [52] in their investigation of BEA-clinoptilolite composites formed by combining BEA-type zeolite with clinoptilolite after alkaline treatment, both with and without ultrasonic irradiation. Sonication reduced the crystallinity of the treated zeolite mixture, resulting in diminished XRD reflection intensities compared to samples processed without ultrasound. Similar observations were reported by Pal et al. [53], who investigated the synthesis of NaP zeolite by sonication. It was shown that increasing ultrasonic irradiation caused a decrease in crystallinity of the obtained zeolite powders without a change in phase purity. Our previous studies also reported reductions in XRD reflection intensity and crystallite size following ultrasonic-assisted alkaline treatment of faujasite and ZSM-5 zeolites [48], [49], [50]. Contrasting outcomes were noted by Roy et al. [54], who synthesized Li-containing Bikitaite zeolite via sonication with variable sonicator power (150–250 W) and irradiation times (3–6 h). Increased ultrasonic power and prolonged sonication promoted zeolite phase formation, yielding more pronounced XRD reflections. Similarly, Vaičiukynienė et al. [55] demonstrated that synthesizing NaA zeolite crystals by sonochemical methods at room temperature and with shorter crystallization periods resulted in higher crystallinity than traditional hydrothermal techniques. Findings similar to [55] were reported by Reinoso et al. [38], who synthesized Na-Y zeolite via ultrasonic pretreatment of gel followed by hydrothermal crystallization under various temperature and time conditions. The discrepancies between these findings and our results likely stem from differences in ultrasonic treatment parameters, such as longer exposure durations, different temperatures, various chemical compositions of prepared gels, and greater sonicator power, which finally influenced both nucleation and crystallization of zeolite products [56], [57], [58].

This research also examines the mechanism of FAU-type zeolite formation under ultrasound, based on available literature. Pal et al. [53] described crystalline zeolite formation from gel precursors containing Si(OH)_4_ and Al(OH)_4_^−^ species that generate zeolite nuclei. At alkaline pH, anionic silicate species dominate, and condensation proceeds in two steps: formation of Si-O-Si bonds from Si(OH)_3_O^−^ groups, followed by water removal and dimer formation. In parallel, Si(OH)_4_ and Al(OH)_4_^−^ condense to form pentacoordinated Si-O-Al intermediates, releasing H_2_O [59]. Ultrasonic irradiation promotes this process by generating H· and OH· radicals during cavitation bubble collapse [60], which contribute to Si–O–Si and Si–O–Al formation. Proton transfer from H_2_O to SiOH through hydrogen bonding also supports mixed aluminosilicate dimer formation, an exothermic process [53], [61]. Cavitation may enhance zeolite synthesis by improving mass transfer through micro-stirring [62], initiating nucleation via localized shock waves [63], creating additional nucleation sites, and accelerating dissolution of silicate and aluminate species. Ultrasound also shifts the depolymerization–polymerization equilibrium by increasing bulk temperature through sound-wave damping [64] and lowering surface Gibbs free energy at the crystal–liquid interface [35]. Together, these effects can shorten aging, accelerate crystallization, and influence crystal size and morphology, often producing more homogeneous crystalline products, though sometimes with broader size and morphology distributions.

**Fig. 2:**
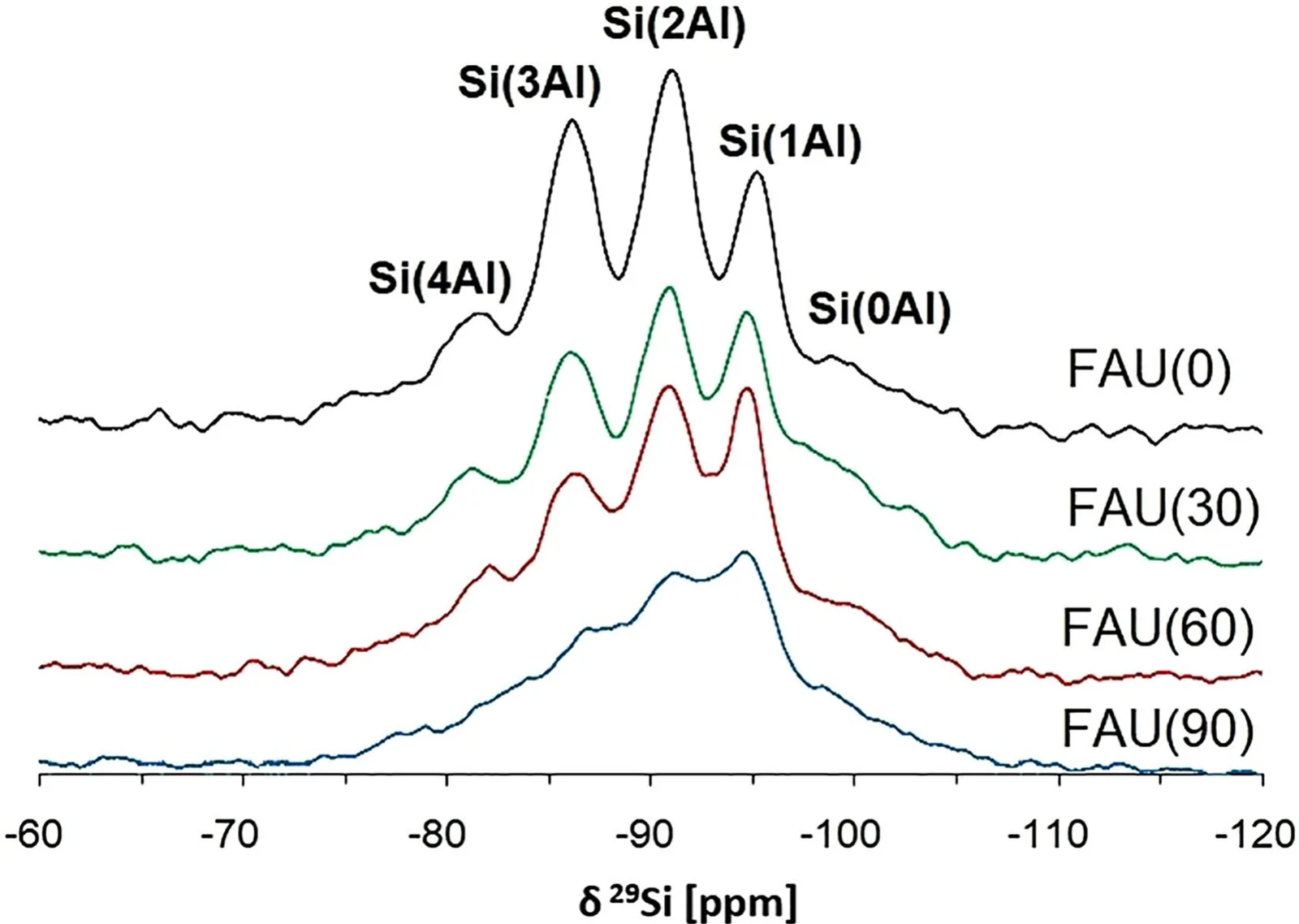
^29^Si MAS NMR spectra under study. The impact of the sonochemical zeolite gel pretreatment time on the chemical environment of silicon in the prepared zeolite systems.

**Fig. 3:**
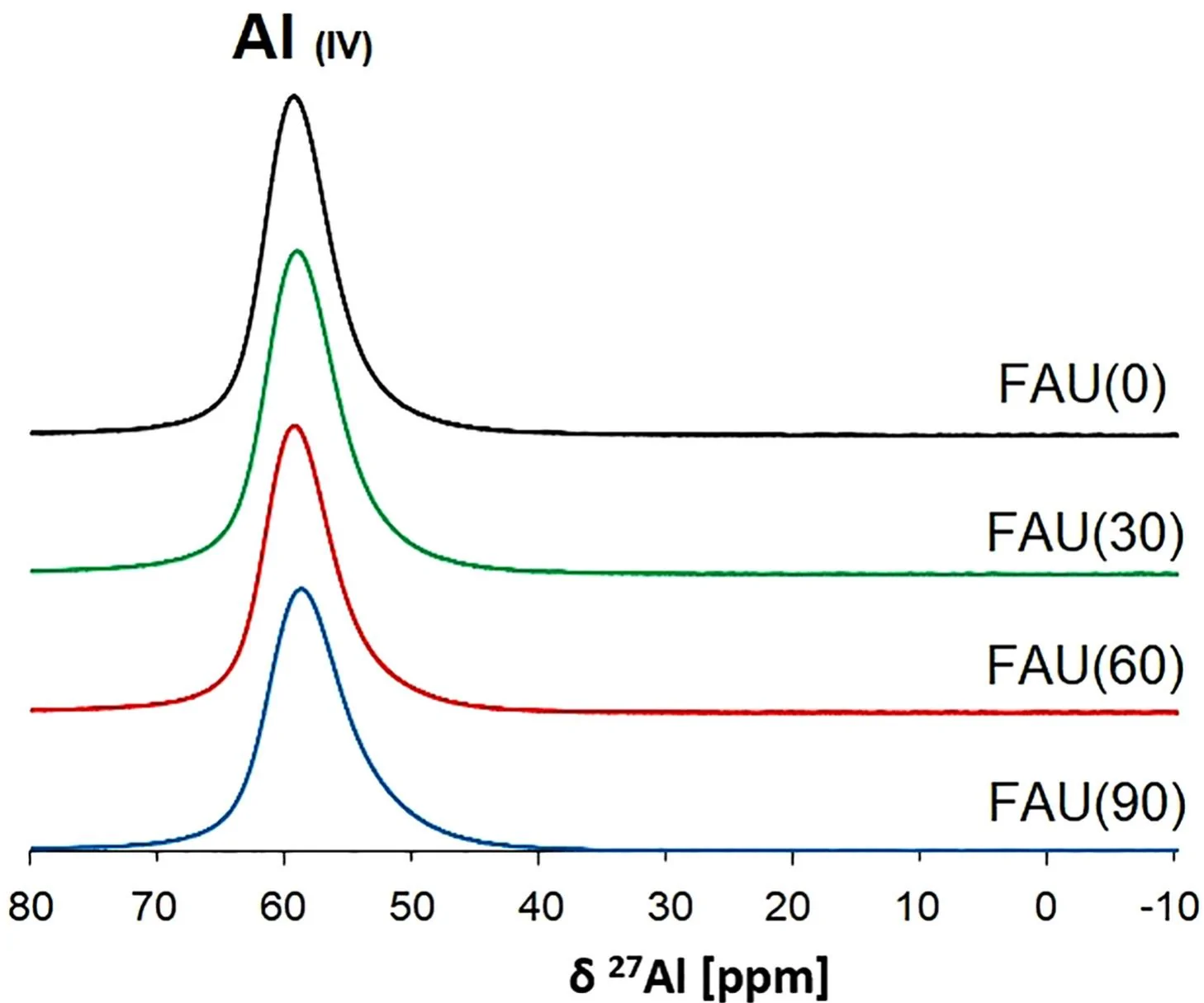
^27^Al NMAS NMR under study. The impact of the time of sonication of the zeolite gel on the chemical form of aluminum in the prepared zeolite samples.

**Fig. 4:**
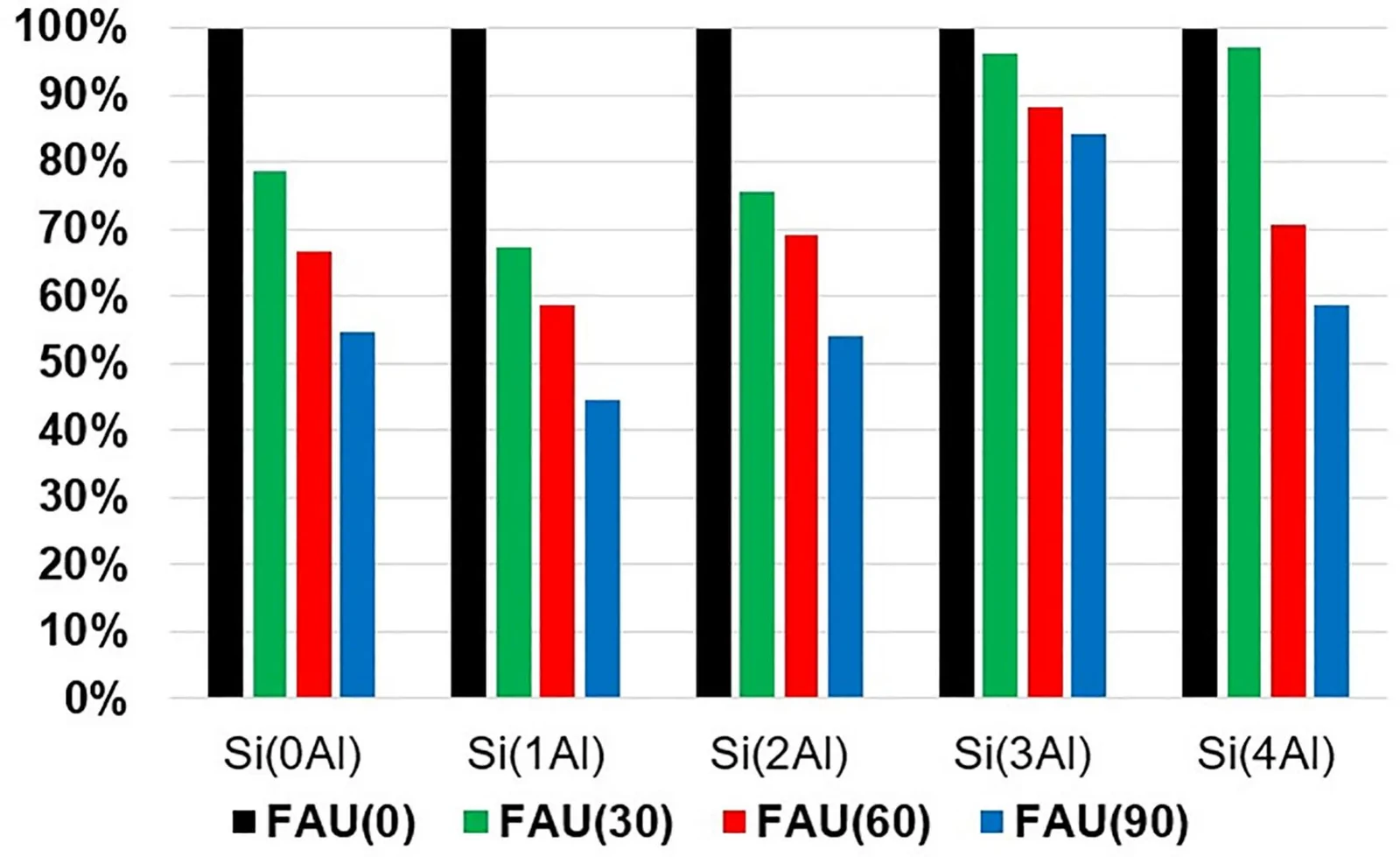
The results of the deconvolution of ^29^Si MAS NMR spectra. Relative decrease of the intensity of the signals assigned to Si(0Al), Si(1Al), Si(2Al), Si(3Al), and Si(4Al). For the reference sample, the original intensity of each signal was marked as 100%.

**Fig. 5:**
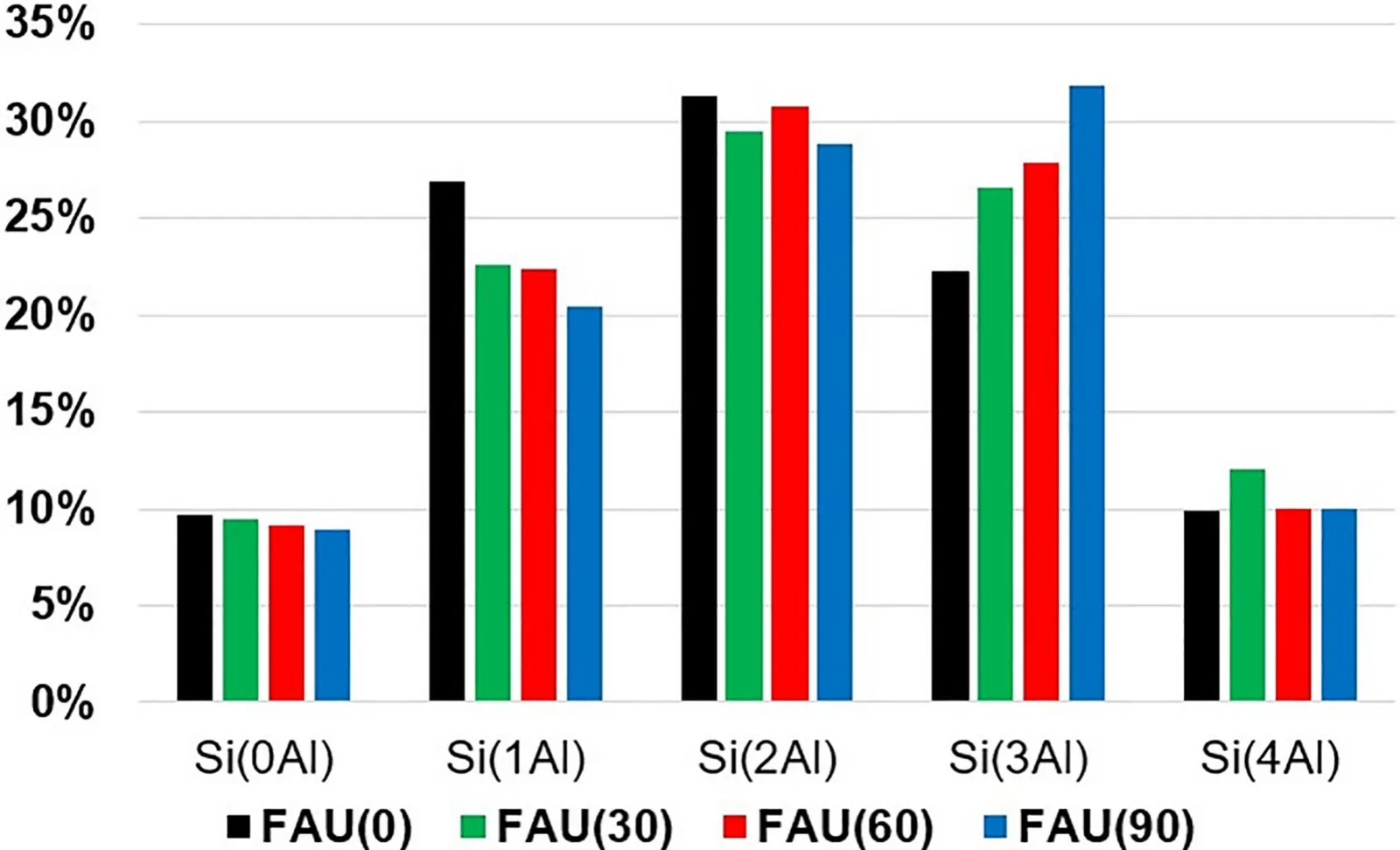
The results of the deconvolution of ^29^Si MAS NMR spectra. Relative contribution of the intensities of signals attributed to Si(0Al), Si(1Al), Si(2Al), Si(3Al), and Si(4Al).

In the present work, ultrasound exposure of the zeolite gel reduced the Si/Al ratio of the crystalline product. The hydrothermal sample, without sonochemistry, exhibited a Si/Al ratio of 2.47, whereas the sonicated samples demonstrated lowered Si/Al ratio values in the range of 1.54–1.86 (Table 1). The scale of the Si/Al drop in the zeolite product correlated with the time of exposure of zeolite gel to ultrasonic irradiation, before a proper hydrothermal synthesis. Interestingly, the direct comparison between Si/Al ratios obtained from the EDS (belonging to surface techniques) and the ICP-OES (being a volumetric method) led to the conclusion that rising sonication time of the zeolite gel favored the crystallization of faujasite, whose grains were enriched or depleted in silicon on the surface or at the depth, respectively. It was also found that the interaction between ultrasound and the gel precursor led to the formation of a zeolite depleted predominantly in silicon and, to a lesser extent, in aluminum, compared with systems not subjected to the sonochemical technique. This hypothesis is supported by ^29^Si and ^27^Al MAS NMR spectra (Fig. 2, Fig. 3), which indicated that ultrasonic treatment generated systems deficient in silicon adjacent to zero, one, two, three, or four aluminum atoms. The quantitative analysis of changes in the silicon population was performed by the deconvolution of ^29^Si MAS NMR spectra, illustrating both a decrease in the intensities of signals attributed to silicon coordinated by various numbers of aluminum atoms (Fig. 4), as well as their relative changes in the contribution between the specific coordination of Si by Al (Fig. 5). The data illustrated in Fig. 4 clearly showed that rising sonication time of the zeolite gel before hydrothermal treatment caused crystallization of zeolites with gradually lower silicon contents, which aligned with the ICP-OES analysis, as well as was in accordance with the observed reduction in crystallinity and crystallite sizes (Table 1, Fig. 1). Additionally, we observed decreasing yields of zeolite products with increasing gel exposure time to ultrasonic irradiation. The weighted mass of sonicated samples corresponded to 96% for FAU(30), 94% for FAU(60), and 80% for FAU(90) in comparison with the yield observed for the reference sample, FAU(0). In turn, the results on the relative contribution of silicon surrounded by various numbers of Al atoms indicated that the population of silicon atoms coordinated by zero or four Al atoms was practically independent of sonication (Fig. 5). Nevertheless, it seems that ultrasonic irradiation of the gel led to the crystallization of faujasite with reduced Si(1Al) and Si(2Al) coordination, as well as elevated Si(3Al) species.

Changes in the chemical composition of the ultrasonically assisted faujasite prepared in this study align with those of hierarchical ZSM-5 systems produced via alkaline treatment under ultrasonic irradiation [50]. Alkaline modification of ZSM-5 resulted in substantial extraction of silicon and aluminum from the zeolite framework, a phenomenon attributed to the presence of an organic base, as confirmed by Sadowska et al. [22], [25] and Abello et al. [65]. In this context, tetrabutylammonium hydroxide interacted with aluminum, facilitating its removal, which was accompanied by the extraction of adjacent silicon atoms. Interestingly, in some cases, the application of ultrasonic irradiation for the preparation of zeolites resulted in higher Si/Al ratios compared with counterparts prepared via standard aging followed by a hydrothermal procedure, as was reported by Reinoso et al. [38] and Zhang et al. [39]. These observations could be explained by the growth in the concentration of soluble silicate species in the presence of ultrasound during gel aging under specific conditions, facilitating their incorporation into the crystallizing zeolite framework [66]. Due to the different aging and crystallization conditions in the current research, it could happen that the selected synthesis parameters led to the formation of silicon species of limited solubility followed by their hindered incorporation into the zeolite structure [57], [58], [66]. Nevertheless, in our case, the drop of the Si/Al ratio for the faujasite powders obtained from sonicated gels in relation to the reference sample can be due to increased depolymerization of Si and Al species in gel solutions as a result of shockwave accompanied by ultrahigh temperature and pressure, followed by the cleavage of Si-O-Si and Si-O-Al bonds in gel [67]. This enables transfer of Si species (to a lesser extent Al species) from the solid to liquid state. It is also possible that the mentioned Si and Al present in the liquid phase do not have enough time to re-incorporate into the zeolite solid phase [35], [60], [61], [62], [63], [64], [65], [66], [67], [68], [69]. Interestingly, despite the loss of crystallinity and microporosity in our sonicated faujasites, no amorphous phase was found. This effect can be explained by the disintegration of the agglomerates by ultrasonic irradiation (so-called sonfractionization). Further washing and centrifugation of the synthesized zeolite sample in water can remove the fragmented and amorphous aluminosilicate phase, leaving only the pure zeolite phase [68], [69].

**Fig. 6:**
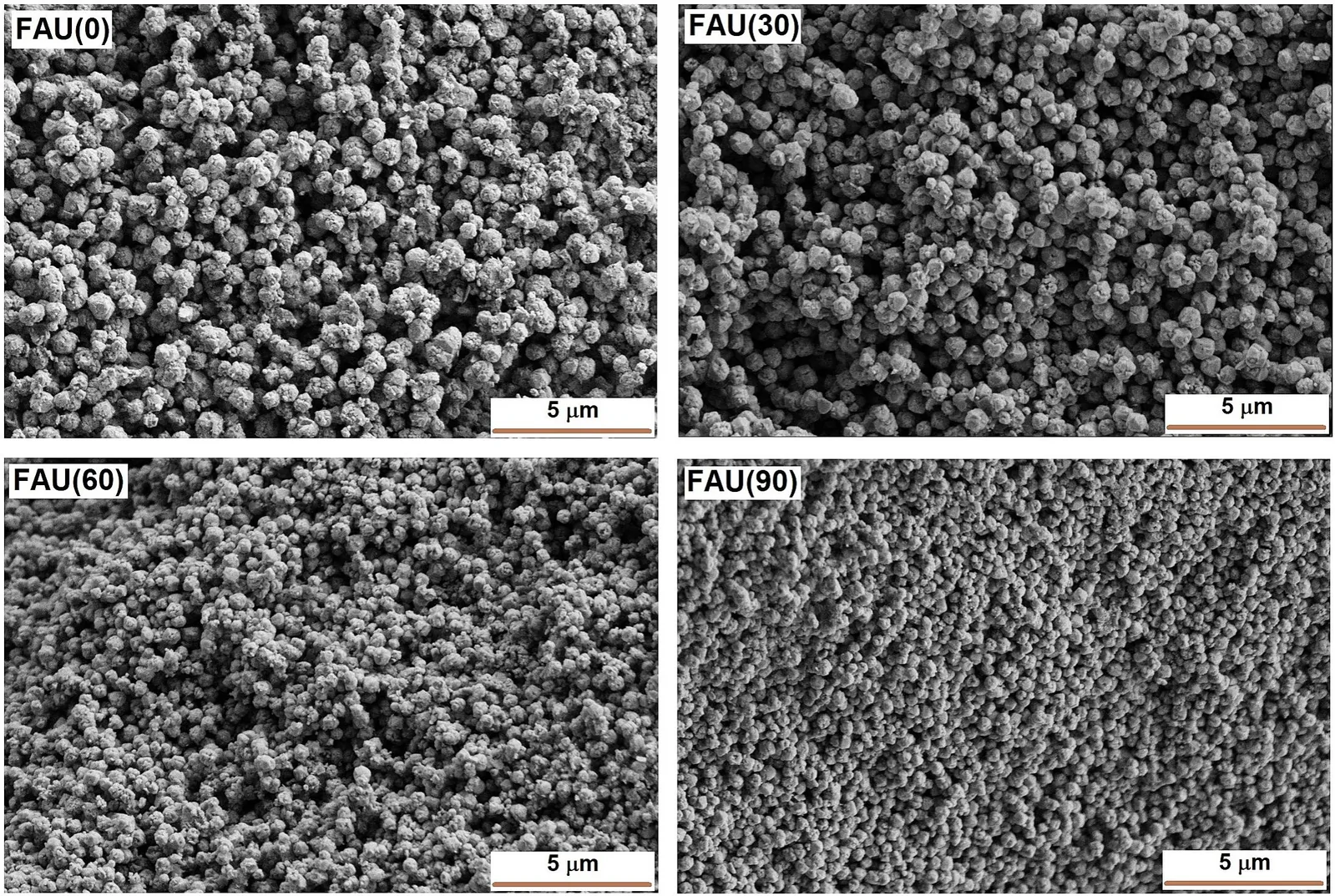
SEM images of the samples under study (magnification 20,000x). The influence of the exposure time of zeolite gel to ultrasonic irradiation on the morphology of the prepared zeolite systems.

**Fig. 7:**
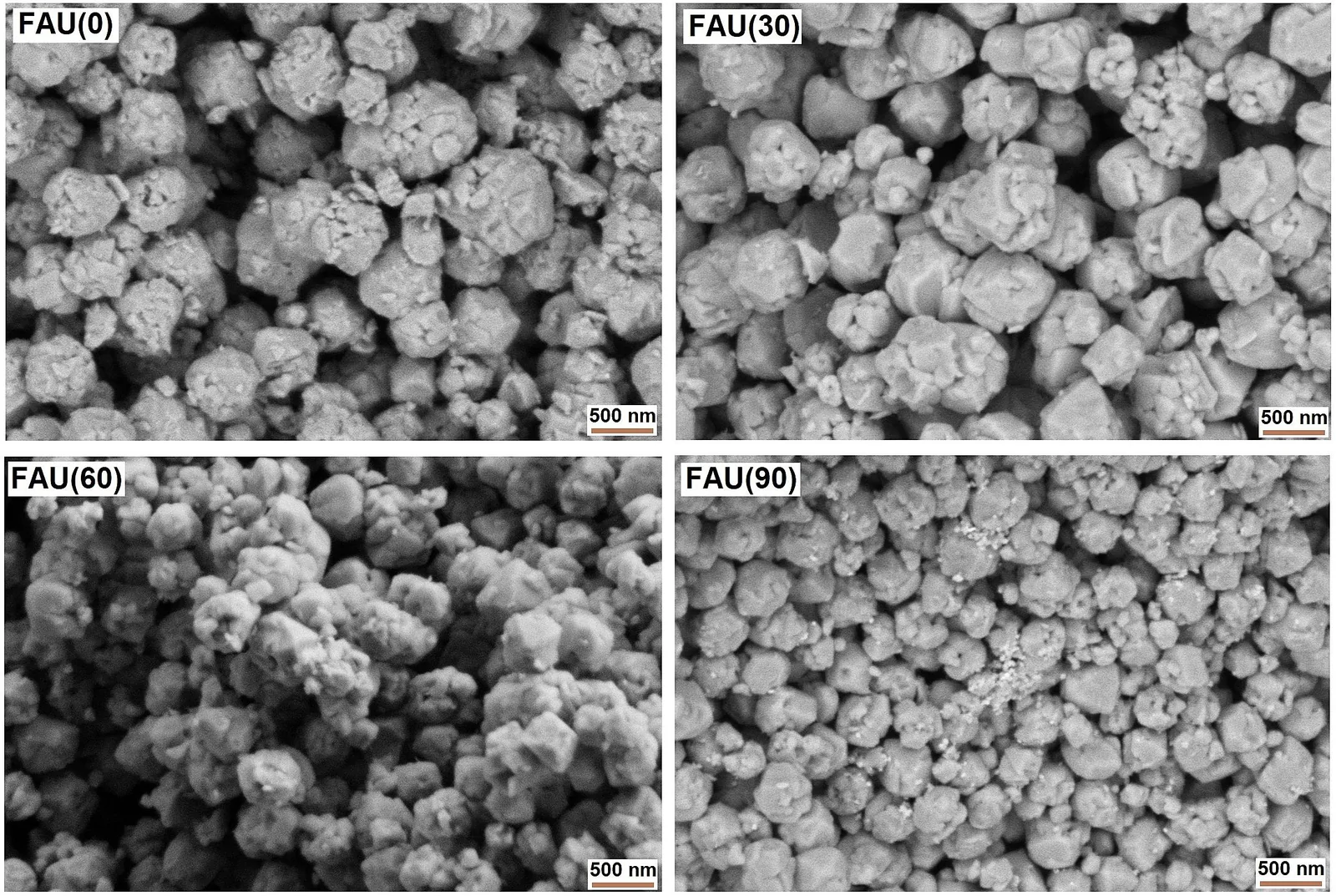
SEM images of the samples under study (magnification 80,000x). The influence of the exposure time of zeolite gel to ultrasonic irradiation on the morphology of the prepared zeolite systems.

**Fig. 8:**
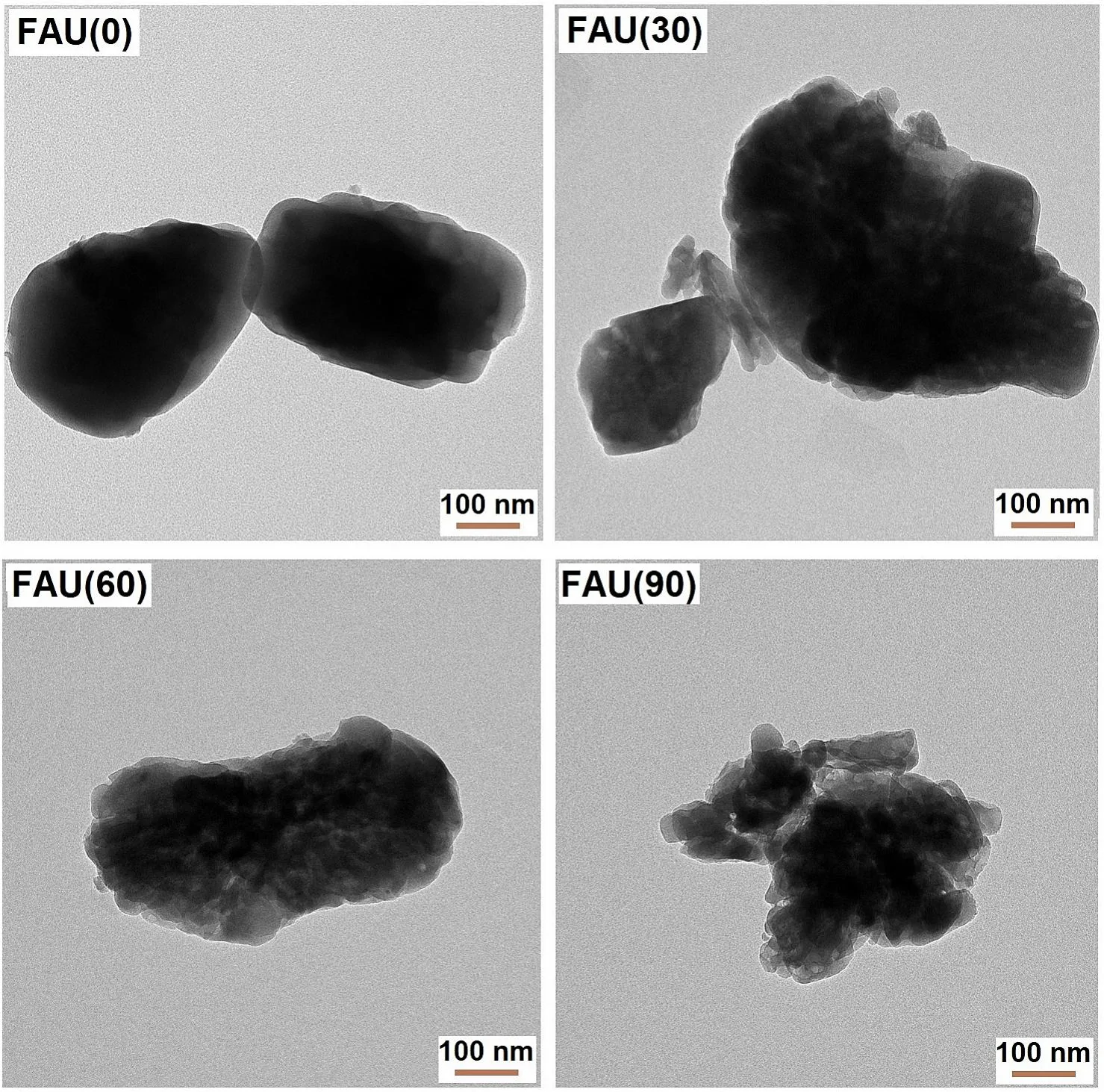
TEM images of the studied samples.

Fig. 6 (magnified to 20,000x) and Fig. 7 (magnified to 80,000x) illustrate the morphological analysis of sonochemically prepared faujasite samples. The SEM images reveal that the zeolite crystals exhibit walnut-like shapes, with sizes ranging from 0.2 to 0.5 µm. Notably, sonication of the gel before hydrothermal crystallization did not induce amorphization in the resulting faujasite product, which is consistent with the absence of ^27^Al and ^29^Si MAS NMR signals associated with Al and Si species in the amorphous phase (see Fig. 2, Fig. 3). Morphological changes attributed to gel sonication followed by hydrothermal treatment were primarily linked to a decrease in zeolite grain size, which corresponded with the reduction of the crystallite sizes calculated from the Scherrer equation (Table 1) and the broadening of XRD signals (Fig. 1). This tendency increased with the sonication time and can be explained by faster nucleation and shorter crystallization time. In other words, a denser localization of seeds (and later crystals) limits their sizes due to the determined volume of the reaction system, which is not infinite [57]. This observation aligns with the research reported by Reinoso et al. [38], who prepared zeolite NaY; Zhang et al. [39], who synthesized zeolite NaX; and Pal et al. [53], who obtained NaP zeolite using ultrasonically assisted aging of gel subjected to further hydrothermal treatment. In turn, more information on the morphology of the prepared faujasite samples can be taken from the appearance of the TEM images (Fig. 8). In the case of samples obtained via hydrothermal crystallization of sonicated gels, the occurrence of grains of pumice-like architecture was found due to the existence of Si-deficient fields observed as bright areas, which constitute the “bitten” structure of zeolite. This limited content of silicon in the faujasites obtained from the ultrasonically-assisted route corresponded to both ICP-OES and ^29^Si MAS NMR analyses (Table 1, Fig. 2, Fig. 3, Fig. 4). A similar pumice-type appearance of zeolite grains was observed by Huang et al. [70], as well as by Kong et al. [71], who synthesized hierarchical systems of ZSM-5-structure type zeolite.

The walnut-shaped morphology of the faujasite grains was in line with observations reported by Yin et al. [72], who synthesized chabazite from zeolite T, as well as aligned with Nemakanga et al. [68], who obtained NaP and NaX zeolites from bentonite. These zeolites were obtained via the ultrasonic-assisted recrystallization of the gel containing the mentioned precursors, subjected to further hydrothermal treatment. The appearance of walnut- and spherical-shaped grains was due to the agglomeration of crystals. A similar effect on morphology was observed by Pal et al. [53] for NaP zeolite, which was characterized by agglomerated particles of cactus / cabbage-shaped grains.

**Fig. 9:**
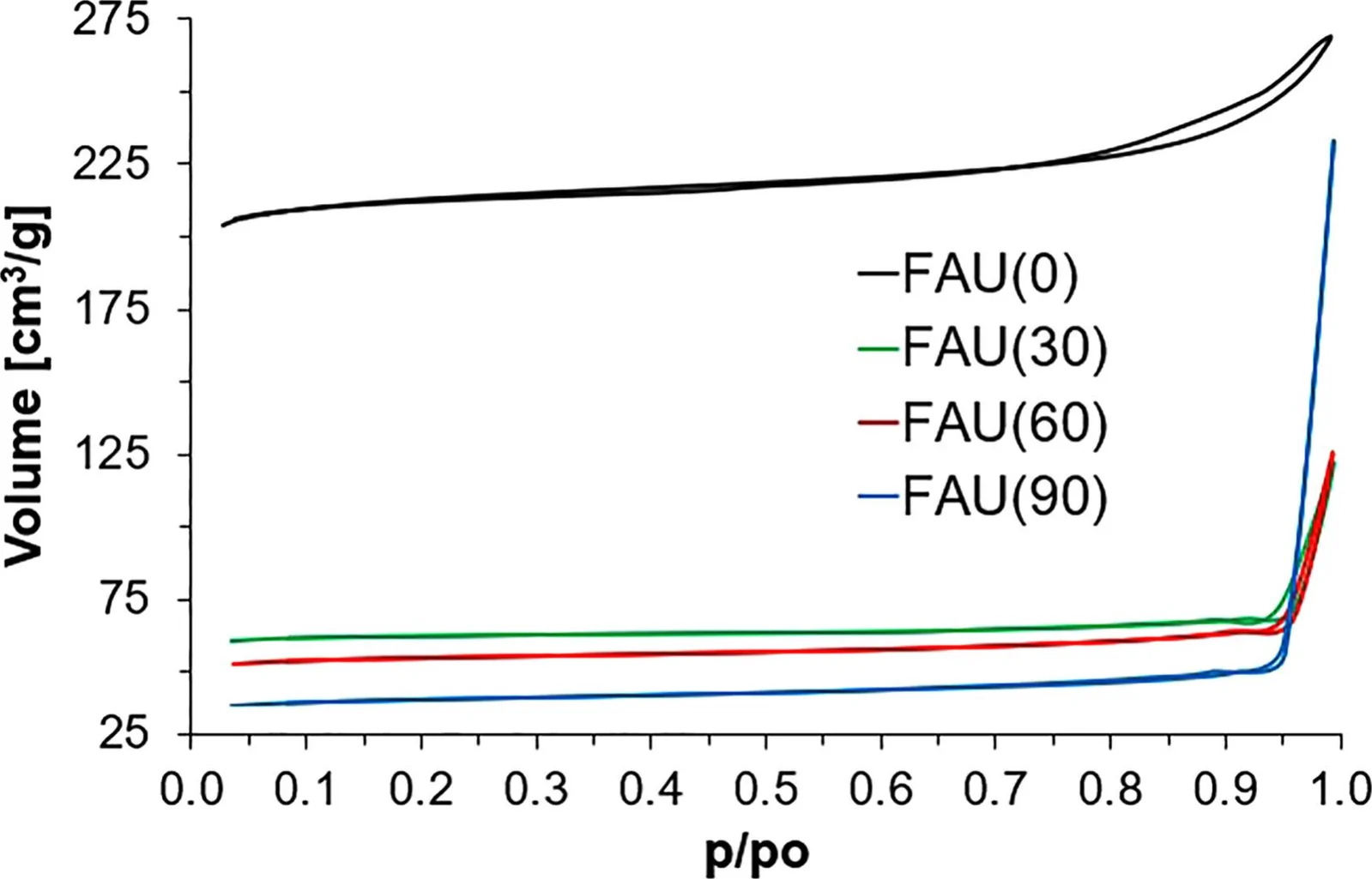
Adsorption-desorption isotherms of nitrogen at −196 °C for the studied samples.

**Fig. 10:**
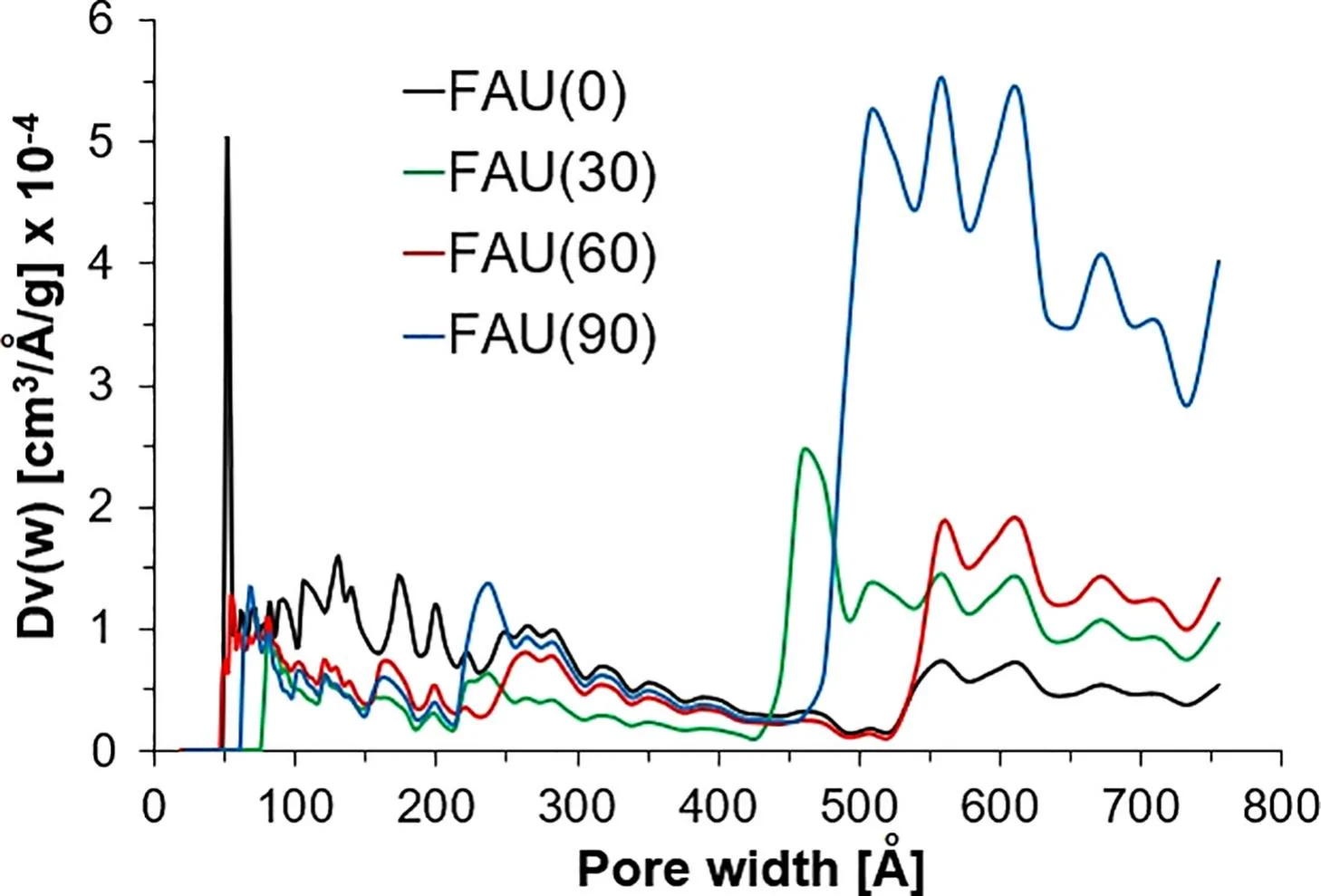
Pore size distributions for the studied samples collected from the DFT model.

The results concerning the porosity of the studied samples are presented in Table 2, as well as in Fig. 9, Fig. 10. At first sight, the results of research on the porosity of FAU-type zeolites, created by using ultrasonic-assisted aging of gels followed by hydrothermal treatment (Table 2), suggested that ultrasound applied during gel aging might produce hierarchical zeolites without needing any post-synthesis modification. Extending the exposure of aged gel to ultrasound gradually led to a marked reduction in micropore volume (from 0.297 to 0.054 cm^3^/g), while simultaneously increasing the seeming meso- and macroporosity (from 29% to 85%).

**Table 2:** The influence of the exposure time of zeolite gel to ultrasound on the porosity of the prepared zeolite systems.

Sample	Porosity			
	S_BET_ [m^2^/g]	V_micro_ [cm^3^/g]	V_total_ [cm^3^/g]	(V_meso_ + V_macro_/V_total_)*100%
FAU(0)	627	0.297	0.416	29
FAU(30)	245	0.092	0.190	52
FAU(60)	212	0.077	0.195	61
FAU(90)	156	0.054	0.361	85

The microporosity loss with the formation of a seeming hierarchical system of pores was confirmed by the appearance of both adsorption–desorption isotherms (Fig. 9) and pore distribution curves based on the DFT model (Fig. 10). For all investigated materials, the occurrence of a hysteresis loop was found. For the reference sample – FAU(0), it can be related to the existence of intercrystalline pores between crystals. This effect was also observed for other microporous zeolite materials, as observed by Verboekend et al. [73], Li et al. [74] and de Jong et al. [75]. The appearance of N_2_ adsorption–desorption isotherms obtained for FAU(0) aligned with IUPAC type I isotherms, with H4 hysteresis, typical for microporous materials. [76]. For the samples prepared using sonication, drastic microporosity collapse accompanied by the weakening and shifting of hysteresis loops in the N_2_ adsorption/desorption isotherms towards higher p/p_o_ values (close to 1) was found. This effect can be observed particularly for the FAU(90) sample. The appearance of these narrow hysteresis loops at 0.95 < p/p_o_ < 1.0 at first sight could be explained by the filling of macropores. The same conclusion might be formulated based on the appearance of pore width distribution profiles obtained from the Density Functional Theory model (Fig. 10). Increasing the time of the gel exposure to ultrasound caused the gradual strengthening of the curve intensity for pore widths higher than 500 Å, which was extremely apparent for the sample obtained from the gel subjected to ultrasonic irradiation for 90 min. Nevertheless, the drop in microporosity observed in the samples (Table 2, Fig. 9, Fig. 10) was associated with the weakened XRD signals (Fig. 1), reflecting lowered crystallinity (Table 1), smaller crystallites (Table 1, Fig. 6, Fig. 7, Fig. 8), Si-deficient faujasite structures, as shown by ^29^Si MAS NMR spectra (Fig. 2, Fig. 3, Fig. 4) and TEM images (Fig. 8). All these observations suggested existing interparticle voids rather than intracrystalline mesopores and macropores. Furthermore, the observed microporosity loss did not lead to the formation of an amorphous phase; there were no NMR signals for amorphous Si or Al species [18], [77], [78], nor was any amorphous phase seen in SEM and TEM images (Fig. 6, Fig. 7, Fig. 8).

Our findings on how ultrasound reduces microporosity align with studies by Orui et al. [44], Zhang et al. [45], Khoshbin et al. [46], [47], and our own previous work [48], [49], [50]. While these references focus on ultrasonic-assisted modifications of microporous zeolites in alkaline conditions after synthesis, it is important to highlight that this study also used sonication of gel precursors in a basic medium before hydrothermal treatment. The consistency between the changes in Si/Al ratios and porous structures of such obtained zeolites can be observed both in our current research and in available literature. As mentioned above, in the present work, the microporosity loss corresponded with the reduction of the Si/Al ratio of sonicated zeolite compared to the reference sample, for which the ultrasonic pretreatment of the gel before hydrothermal crystallization was omitted. Analogously, in the research reported by Reinoso et al. [38] and Zhang et al. [39], higher Si/Al ratios were correlated with higher textural parameters, particularly those related to microporosity.

Concerning the porosity, the observed formation of seemingly hierarchical faujasites, obtained in the presence of ultrasound in the present work, can be explained by the behavior of cavitation bubbles as transient “soft templates”. Due to their periodic growth and collapse, the occurring localized mechanical stress can reopen blocked channels and pores, finally leading to the generation of interparticle voids [79]. Such multiscale porosity improves pore connectivity and mass diffusion, leading to greater utilization of catalytic sites [80].

Acidity studies employing NH_3_-IR revealed that the utilization of ultrasound during the synthesis and modification of FAU-type zeolites resulted in decreased concentrations of both Brønsted and Lewis acid sites. These reductions correlated with increased exposure time to ultrasound, ranging from 0 to 90 min. Specifically, Brønsted and Lewis acid site concentrations decreased from 151 μmol/g to 61 μmol/g and from 272 μmol/g to 60 μmol/g, respectively (Table 3). The observed reduction in acidity was analogous to the effect noted for hierarchical zeolite of ZSM-5 structure obtained via ultrasonic-assisted alkaline treatment [50]. Lower levels of both Brønsted and Lewis acid sites were attributed to the extraction of silicon and aluminum, consistent with findings from the ^27^Al and ^29^Si MAS NMR spectra (Fig. 2, Fig. 3), as well as TEM images (Fig. 8). It must be pointed out that, based on the absence of signals at 0 ppm in the ^27^Al MAS NMR spectra (Fig. 3), no formation of extra-framework Al species was found as a result of the sonochemical treatment of the gel from which the zeolite was crystallized. This means that no production of Lewis acidity at the expense of protonic acid sites occurred. Another reason for which the reduced Brønsted and Lewis concentrations were found was the increasing surface Si/Al ratio determined by EDS analysis (Table 1), which could slightly hinder access to Al for ammonia.

**Table 3:** The impact of the ultrasonic pretreatment duration of zeolite gel on the acidity of the prepared zeolite systems.

Sample	FAU(0)		FAU(30)		FAU(60)		FAU(90)	
Temperature	BAS* [μmol/g]	LAS* [μmol/g]	BAS* [μmol/g]	LAS* [μmol/g]	BAS* [μmol/g]	LAS* [μmol/g]	BAS* [μmol/g]	LAS* [μmol/g]
120 °C	151	272	135	236	132	164	61	60
140 °C	121	363	87	289	75	191	26	74
160 °C	91	151	77	243	36	106	6	11
180 °C	60	92	58	152	9	21	traces	traces
200 °C	23	27	29	30	traces	traces	traces	traces
240 °C	traces	traces	traces	traces	traces	traces	traces	traces

*BAS − Brønsted Acid Sites, *LAS – Lewis Acid Sites.

These results align with those reported by Solyman et al. [29], who investigated the acidity of BEA and H-Mordenite zeolites after various durations of sonication. In mordenite, ultrasound application led to a reduction in all acid site types. A comparable effect was observed in BEA zeolites; however, mordenite samples retained a more acidic profile than their BEA counterparts.

In turn, the quantitative analysis of the FT-IR-monitored ammonia sorption conducted in the temperature range of 120 °C- 240 °C (Table 3) indicated a weak acidic strength of the studied materials. For all studied samples, all ammonia was desorbed at 240 °C. It must be underlined that in all cases, the growth of NH_3_ desorption temperature from 120 °C to 140 °C led to an apparent increase in the concentration of Lewis acid sites at the expense of Brønsted acidity. This phenomenon can be explained by partial readsorption of ammonia on stronger Lewis acid sites, whose molecules were just desorbed from weaker protonic counterparts [81], [82]. Further heating of the studied samples led to more pronounced desorption of ammonia from both acid-site types. Nevertheless, it seems that protonic acid site concentrations dropped more slowly with NH_3_ sorption temperature than those of Lewis sites, which confirmed a higher strength of Brønsted acidity in comparison with the Lewis-type acid sites [83], [84]. The observed weak acidity of the studied zeolites can be attributed to very low Si/Al ratios (ranging from 1.54 to 2.47; see Table 1). As is commonly known, acidity strength depends on the Si/Al ratio. Higher Si/Al ratios correspond to higher acidity strength [85], [86].

Our ultrasonically prepared zeolites were inactive in the studied reaction, decarbonylation of furfural to furan. For all samples, the interaction between the zeolite framework and the gaseous reaction mixture led to the collapse of the zeolite crystallinity (Fig. S1). Catalytic inactivity can also be explained by the low acid strength of the prepared catalysts (Table 3). In the tested reaction temperature region (at 250–350 °C), the population of strong acid sites able to take part in the studied reaction was negligible. Low acid strength is typical of zeolites with very low Si/Al ratios (for sonicated samples, these values are lower than 2). Further studies will include more siliceous zeolite-type structures of higher starting Si/Al ratios and higher acid strength, e.g., ZSM-5, MOR, or BEA. It is also expected that those structures obtained via sonication would still maintain high Si/Al ratios with preserved crystallinity.

### 3.2 Green and economic aspects of sonication

Ultrasonic technology is widely recognized as a green and efficient process-intensification method for material synthesis. Therefore, compared with our previous research devoted to ultrasonic-assisted alkaline treatment [48], [49], [50], the present work represents a step forward: the synthesis of zeolites with a faujasite-type structure, using ultrasound as an alternative method for desilication, was developed. This action enabled the elimination of the use of expensive reagents (such as tetraalkylammonium hydroxides (TBAOH) while also protecting the natural environment (Table S1). The cost of one small bottle of TBAOH is higher than the fare for the energy consumed during the work of the sonicator for even 90 min [87], [88]. Generally, the preparation of zeolite via ultrasonic-assisted aging of gel followed by hydrothermal treatment seems to be much cheaper than desilication of microporous zeolites via post-synthesis alkaline treatment using NaOH and tetraalkylammonium hydroxides.

In the context of ultrasonic-assisted synthesis of organic template-free zeolite systems, our research can be compared with the available literature. For instance, Boffito et al. [30] compared ultrasonic-assisted hydrothermal synthesis, direct ultrasonic synthesis without a hydrothermal step, and mechanically stirred hydrothermal treatment for template-free zeolite A production. Direct sonochemical synthesis reduced the total synthesis time from 11 h to 6 h, increased the yield by 60%, and eliminated the need for subsequent hydrothermal treatment. In turn, Yin et al. [36] showed that an ultrasonic-assisted hydrothermal route enabled template-free synthesis of chabazite-type zeolite and reduced CHA crystallization from 48 h to 10 h. Regarding temperature, Belviso et al. [31] found that ultrasonic irradiation enabled low-silica A- and X-type zeolites to crystallize at lower temperatures, between 25 and 60 °C, using feedstocks such as fly ash and seawater.

For other zeolite systems, Chew et al. [27] combined ultrasonic irradiation with hydrothermal treatment to prepare RHO zeolite, reducing the synthesis time from 7 days to 2 days relative to conventional hydrothermal synthesis. Yeong et al. [28] reported a similar effect: a 60 min sonochemical pre-treatment shortened zeolite T synthesis from 7 days to 1 day. Also, in one of our previous works [42], we developed Cu- or Co– or Fe-containing BEA-type zeolites as catalysts for the production of acrylic acid from lactic acid using a standard ion-exchange procedure and ultrasonic irradiation. It was found that ultrasonic-assisted deposition of Cu or Co or Fe on a zeolite carrier for 20 min was more effective than the ion-exchange procedure for 24 h. Furthermore, the highest selectivities to acrylic acid were found predominantly for the catalysts obtained sonically due to a better distribution of the metallic active phase and its chemical form as M−O−M groups, which were the most responsible for the formation of acrylic acid from lactic acid.

## 4 Summary and conclusions

Compared to ultrasonic-assisted alkaline treatment, the synthesis of hierarchical zeolites with a faujasite-type structure could be advanced by employing ultrasound as an alternative method for desilication. This approach could eliminate the need for costly and hazardous reagents, such as tetraalkylammonium hydroxides, thereby contributing to environmental protection. The initial stage of synthesis involved preparing zeolite gels, which underwent aging followed by ultrasonic pretreatment for a specified duration. Subsequent hydrothermal processing enabled the crystallization of the final zeolite product. A reference sample was produced without ultrasound exposure.

Increasing the ultrasound exposure time of zeolite gels led to systems with smaller crystallites and reduced concentrations of Brønsted and Lewis acid sites, similar to effects observed in hierarchical zeolites of faujasite or ZSM-5 structure types prepared via ultrasonic-assisted alkaline treatment. The lower concentrations of both acid site types and their lowered acid strength for the sonicated samples in comparison with the parent sample were linked to the losses in microporosity and specific surface area attributed to Si-deficient regions in zeolite structures, as well as to a possible migration of Si from the depth of the zeolite grain to its surface, which hindered the access of Al-related acid sites to the ammonia probe molecule. These findings were corroborated by NMR spectra, chemical analyses, and TEM images. On the other hand, significant changes in porosity, accompanied by distinct fragmentation and a relatively low drop in Si/Al ratios, suggested the presence of interparticle voids rather than intracrystalline mesopores and macropores. Therefore, the sonicated materials reported in this work can be described as seemingly hierarchical faujasites. The sonicated faujasites exhibited high sensitivity to further modification, therefore, future studies will investigate zeolite structures of higher starting Si/Al ratios, including MFI, BEA, and MOR. Hence, this method requires further optimization and investigation in the context of their catalytic applications.

## CRediT authorship contribution statement

**Łukasz Kuterasiński:** Writing – review & editing, Writing – original draft, Visualization, Validation, Supervision, Software, Resources, Project administration, Methodology, Investigation, Funding acquisition, Formal analysis, Data curation, Conceptualization. **Małgorzata Ruggiero-Mikołajczyk:** Methodology, Investigation, Formal analysis. **Małgorzata Zimowska:** Methodology, Investigation, Formal analysis. **Mariusz Gackowski:** Writing – review & editing, Methodology, Investigation, Formal analysis. **Grzegorz Kurowski:** Investigation, Formal analysis. **Przemysław Jakub Jodłowski:** Investigation, Formal analysis.

## Declaration of competing interest

The authors declare that they have no known competing financial interests or personal relationships that could have appeared to influence the work reported in this paper.

## Appendix A Supplementary data

Supplementary Data 1 XRD patterns of spent catalysts & Economic aspects of sonication.

## References

[1] Mao J. (2025). Proceedings of the 3rd International Conference on Mechatronics and Smart Systems.

[2] https://www.iza-structure.org/databases/ (assess on 23.07.2026).

[3] Pavlović J., Rajić N. (2026). Engineering zeolite acidity and porosity for improved esterification: a review of mechanisms, kinetics, and sustainability processes. Minerals.

[4] Ferrarelli G., Migliori M., Catizzone E. (2024). Recent trends in tailoring external acidity in zeolites for catalysis. ACS Omega.

[5] Bajec D., Veryasov G. (2026). Prospective future of zeolites – from molecular to systems level. Catal. Today.

[6] Zhang M., Huang J., Meng F., Zhang C., Zhang Z. (2025). Coke-induced deactivation in zeolite catalysts: mechanisms, anti-coking modifications, and regeneration approaches. Dalton Trans..

[7] van Donk S., Bitter J.H., Verberckmoes A., Versluijs-Helder M., Broersma A., de Jong K.P. (2005). Physicochemical characterization of porous materials: spatially resolved accessibility of zeolite crystals. Angew. Chem. Int. Ed..

[8] Pérez-Ramírez J., Christensen C.H., Egeblad E., Christensen C.H., Groen J.C. (2008). Hierarchical zeolites: enhanced utilization of microporous crystals in catalysis by advances in materials design. Chem. Soc. Rev..

[9] Groen J.C., Moulijn J.A., Perez-Ramirez J. (2006). Desilication: on the controlled generation of mesoporosity in MFI zeolites. J. Mater. Chem..

[10] Zhao L., Shen B., Gao J., Xu C. (2008). Investigation on the mechanism of diffusion in mesopore structured ZSM-5 and improved heavy oil conversion. J. Catal..

[11] Gopalakrishnan S., Zampieri A., Schwieger W. (2008). Mesoporous ZSM-5 zeolites via alkali treatment for the direct hydroxylation of benzene to phenol with N_2_O. J. Catal..

[12] Egeblad K., Christensen C.H., Kustova M., Christensen C.H. (2008). Templating mesoporous zeolites. Chem. Mater..

[13] Chal R., Grardin C., Bulut M., van Donk S. (2011). Overview and industrial assessment of synthesis strategies towards zeolites with mesopores. ChemCatChem.

[14] Groen J.C., Jansen J.C., Moulijn J.A., Perez-Ramirez J. (2004). Optimal aluminum-assisted mesoporosity development in MFI zeolites by desilication. Phys. Chem..

[15] Jin F., Cui Y., Li Y. (2008). Effect of alkaline and atom-planting treatment on the catalytic performance of ZSM-5 catalyst in pyridine and picolines synthesis. Appl. Catal. A.

[16] Pérez-Ramírez J., Verboekend D., Bonilla A., Abelló S. (2009). Zeolite catalysts with tunable hierarchy factor by pore-growth moderators. Adv. Funct. Mater..

[17] Verboekend D., Pérez-Ramírez J. (2011). Desilication mechanism revisited: highly mesoporous all-silica zeolites enabled through pore-directing agents. Chem Eur J.

[18] Van Aelst J., Haouas M., Gobechiya E., Houthoofd K., Philippaerts A., Sree S.P., Kirschhock C.E.A., Jacobs P., Martens J.A., Sels B.F., Taulelle F. (2014). Hierarchization of USY Zeolite by NH_4_OH. A postsynthetic process investigated by NMR and XRD. J. Phys. Chem. C.

[19] Verboekend D., Nuttens N., Locus R., Van Aelst J., Verolme P., Groen J.C., Pérez-Ramírez J. (2016). Synthesis characterisation, and catalytic evaluation of hierarchical faujasite zeolites: milestones, challenges, and future directions. Sels Chem. Soc. Rev..

[20] García J.R., Falco M., Sedran U. (2016). Impact of the desilication treatment of Y zeolite on the catalytic cracking of bulky hydrocarbon molecules. Top. Catal..

[21] Kim J., Choi M., Ryoo R. (2010). Effect of mesoporosity against the deactivation of MFI zeolite catalyst during the methanol-to-hydrocarbon conversion process. J. Catal..

[22] Sadowska K., Góra-Marek K., Datka J. (2012). Hierarchic zeolites: acid properties of zeolite ZSM-5 desilicated with NaOH and NaOH/tetrabutylamine hydroxide studied by IR spectroscopy. Vib. Spectr..

[23] Verboekend D., Vilé G., Pérez-Ramírez J. (2012). Mesopore formation in USY and beta zeolites by base leaching: selection criteria and optimization of pore-directing agents. Cryst. Growth Des..

[24] Groen J.C., Sano T., Moulijn J.A., Pérez-Ramírez J. (2007). Alkaline-mediated mesoporous mordenite zeolites for acid-catalyzed conversions. J. Catal..

[25] Sadowska K., Góra-Marek K., Drozdek M., Kuśtrowski P., Datka J. (2013). Desilication of highly siliceous zeolite ZSM-5 with NaOH and NaOH/tetrabutylamine hydroxide. Microporous Mesoporous Mater..

[26] Mlekodaj K., Tarach K., Datka J., Gora-Marek K., Makowski W. (2014). Porosity and accessibility of acid sites in desilicated ZSM-5 zeolites studied using adsorption of probe molecules. Microporous Mesoporous Mater..

[27] Ng T.Y.S., Chew T.L., Yeong Y.F. (2019). Synthesis of small pore zeolite via ultrasonic-assisted hydrothermal synthesis. Mater. Today Proc..

[28] Jusoh N., Yeong Y.F., Mohamad M., Lau K.K., Sharif A.M. (2017). Rapid-synthesis of zeolite T via sonochemical-assisted hydrothermal growth method. Ultrason. Sonochem..

[29] Solyman S.M., Aboul-Gheit N.A.K., Tawfik F.M., Sadek M., Ahmed H.A. (2013). Performance of ultrasonic-treated nano-zeolites employed in the preparation of dimethyl ether. Egypt. J. Pet..

[30] Braide D., Al-Sakkari E.G., Panaritis C., Li L.Z., Etienne N.K., Lavigne L., Abanobi A., Ragab A., Patience G., Boffito D.C. (2025). Ultrasound accelerates zeolite synthesis: Predictive modelling via machine learning. J. Environ. Chem. Eng..

[31] Belviso C., Cavalcante F., Fiore S. (2013). Ultrasonic waves induce rapid zeolite synthesis in a seawater solution. Ultrason. Sonochem..

[32] Ran J., Sun X., Zhao J., Wei S., Duan H., Chen Y., Zhang L., Yin S. (2026). Ultrasonic-enhanced Cu(I)/Cu(II) nanointerfaces for sustainable ozone activation in green aluminum production: atomic-level catalysis of organic waste degradation. Green Energy Environ..

[33] Sen M., Dana K., Das N. (2018). Development of LTA zeolite membrane from clay by sonication-assisted method at room temperature for H_2_-CO_2_ and CO_2_-CH_4_ separation. Ultrason. Sonochem..

[34] Andac O., Tatlıer M., Sirkecioglu A., Ece I., Erdem-Senatalar A. (2005). Effects of ultrasound on zeolite a synthesis. Microporous Mesoporous Mater..

[35] Wang B., Wu J., Yuan Z.Y., Li N., Xiang S. (2008). Synthesis of MCM-22 zeolite by an ultrasonic-assisted aging procedure. Ultrason. Sonochem..

[36] Yin X., Long Z., Wang C., Li Z., Zhao M., Yang S. (2019). A time- and cost-effective synthesis of CHA zeolite with small size using ultrasonic-assisted method. Ultrason. Sonochem..

[37] Mu Y., Zhang Y., Fan J., Guo C. (2017). Effect of ultrasound pretreatment on the hydrothermal synthesis of SSZ-13 zeolite. Ultrason. Sonochem..

[38] Reinoso D., Adrover M., Pedernera M. (2017). Green synthesis of nanocrystalline faujasite zeolite. Ultrason. Sonochem..

[39] Zhang X., Li S., Zhang G. (2019). Synthesis of small crystals zeolite NaX by an ultrasonic aging method. Dig. J. Nanomater. Biostruct..

[40] Jodłowski P., Kuterasiński Ł., Jędrzejczyk R.J., Chlebda D., Gancarczyk A., Basąg S., Chmielarz L. (2017). DeNO_x_ abatement modelling over sonically prepared copper USY and ZSM5 structured catalysts. Catalysts.

[41] Kuterasiński Ł., Wojtkiewicz A., Kurowski G., Jeleń P., Sitarz M., Ruggiero-Mikołajczyk M., Gackowski M., Jodłowski P.J. (2025). Ultrasonic-assisted impregnation as an efficient tool for the manufacture of Cu-containing faujasite as an active catalyst for the oxidation of cyclohexene. ACS Omega.

[42] Sobuś N., Michorczyk B., Piotrowski M., Kuterasiński Ł., Chlebda D.K., Łojewska J., Jędrzejczyk R.J., Jodłowski P., Piotr Kuśtrowski I. (2019). Czekaj „Design of Co, Cu and Fe-BEA zeolite catalysts for selective conversion of lactic acid into acrylic acid”. Catal. Lett..

[43] Chlebda D.K., Stachurska P., Jędrzejczyk R.J., Kuterasiński Ł., Dziedzicka A., Górecka S., Chmielarz L., Łojewska J., Starz S., Jodłowski P.J. (2018). NO_x_ abatement over sonically prepared iron substituted Y, USY and MFI zeolite catalysts in lean exhaust gas conditions”. Nanomaterials.

[44] Oruji S., Khoshbin R., Karimzadeh R. (2018). Preparation of hierarchical structure of Y zeolite with ultrasonic-assisted alkaline treatment method used in catalytic cracking of middle distillate cut: the effect of irradiation time. Fuel Process Technol..

[45] Zhang R., Zhong P., Arandiyan H., Guan Y., Liu J., Wang N., Jiao Y., Fan X. (2020). Using ultrasound to improve the sequential post-synthesis modification method for making mesoporous Y zeolites. Front. Chem. Sci. Eng..

[46] Khoshbin R., Karimzadeh R. (2017). Synthesis of mesoporous ZSM-5 from rice husk ash with ultrasound-assisted alkali-treatment method used in catalytic cracking of light naphtha. Adv. Powder Technol..

[47] Khoshbin R., Oruji S., Karimzadeh R. (2018). Catalytic cracking of light naphtha over hierarchical ZSM-5 using rice husk ash as silica source in presence of ultrasound energy: effect of carbon nanotube content. Adv. Powder Technol..

[48] Kuterasiński Ł., Rojek W., Gackowski M., Zimowska M., Jodłowski P.J. (2020). Sonically modified hierarchical FAU-type zeolites as active catalysts for the production of furan from furfural. Ultrason. Sonochem..

[49] Kuterasiński Ł., Filek U., Zimowska M., Napruszewska B.D., Gackowski M., Jodłowski P.J. (2022). Ultrasonically prepared mesoporous zeolites with faujasite-type structure as catalysts for the production of diethyl ether and ethylene from ethanol. Mater. Res. Bull..

[50] Kuterasiński Ł., Filek U., Gackowski M., Zimowska M., Ruggiero-Mikołajczyk M., Jodłowski P.J. (2021). Sonochemically prepared hierarchical MFI-type zeolites as active catalysts for catalytic ethanol dehydration. Ultrason. Sonochem..

[51] D.W. Breck. U.S. Patent No. 3 130 007 (1964).

[52] Safari S., Khoshbin R., Karimzadeh R. (2019). Beneficial use of ultrasound irradiation in synthesis of beta–clinoptilolite composite used in heavy oil upgrading process. RSC Adv..

[53] Pal P., Das J.K., Das N., Bandyopadhyay S. (2013). Synthesis of NaP zeolite at room temperature and short crystallization time by sonochemical method. Ultrason. Sonochem..

[54] Roy P., Das N. (2017). Ultrasonic-assisted synthesis of Bikitaite zeolite: a potential material for hydrogen storage application. Ultrason. Sonochem..

[55] Vaičiukynienė D., Kantautas A., Vaitkevičius V., Jakevičius L., Rudžionis Ž., Paškevičius M. (2015). Effects of ultrasonic treatment on zeolite NaA synthesized from by-product silica. Ultrason. Sonochem..

[56] Mendoza H.R., Jordens J., Pereira M.V.L., Lutz C., Van Gerven T. (2020). Effects of ultrasonic irradiation on crystallization kinetics, morphological and structural properties of zeolite FAU. Ultrason. Sonochem..

[57] Mallette A.J., Shilpa K., Rime J.D. (2024). The current understanding of mechanistic pathways in zeolite crystallization. Chem. Rev..

[58] S. Valange, G. Hatel, P.N. Amaniampong, R. Behling, F. Jérôme “Ultrasound-Assisted Synthesis of Nanostructured Oxide Materials: Basic Concepts and Applications to Energy” in “Advanced solid catalysts for renewable energy production” (S. Gonzalez-Cortes and F. E. Imbert, editors.) (2018), p. 177. doi:10.4018/978-1-5225-3903-2.ch007.

[59] Trinh T.T., Jansen A.P., van Santen R.A. (2006). Mechanism of oligomerization reactions of silica. J. Phys. Chem. B.

[60] Augugliaro V., Litter M., Palmisano L., Soria J. (2006). The combination of heterogeneous photocatalysis with chemical and physical operations: a tool for improving the photoprocess performance. J. Photochem. Photobiol. C.

[61] Catlow C.R.A., Coombes D.S., Lewis D.W., Pereira J.G.C. (1998). Computer modeling of nucleation, growth, and templating in hydrothermal synthesis. Chem. Mater..

[62] Boels L., Wagterveld R.M., Mayer M.J., Witkamp G.J. (2010). Seeded calcite sonocrystallization. J. Cryst. Growth.

[63] Guo Z., Zhang M., Li H., Wang J., Kougoulos E. (2005). Effect of ultrasound on anti-solvent crystallization process. J. Cryst. Growth.

[64] Horst C., Gogate P.R., Pandit A.B. (2007). Ultrasound Reactors, Modeling of Process Intensification.

[65] Abello S., Bonilla A., Perez-Ramirez J. (2009). Mesoporous ZSM-5 zeolite catalysts prepared by desilication with organic hydroxides and comparison with NaOH leaching. Appl. Catal. A. Gen..

[66] Askari S., Alipour S.M., Halladj R., Farahani M.H.D.A. (2013). Effects of ultrasound on the synthesis of zeolites: a review. J. Porous Mater..

[67] Vaiciukyniene D., Jakevicius L., Kantautas A., Vaitkevicius V., Vaiciukynas V. (2020). The effect of ultrasound on Na-A zeolite synthesis based on sodium aluminosilicate gels and solid materials. Rev. Rom. Mater..

[68] Nemakanga S., Ayinde W.B., Mudzielwana R. (2025). Ultrasound-assisted synthesis of zeolite from bentonite clay: optimization using response surface methodology. Bull. Mater. Sci..

[69] Nzodom W., Chave T., Valange S., Nikitenko S.I. (2024). Sonohydrothermal synthesis of zeolite A and its phase transformation to sodalite. Dalton Trans..

[70] Huang L., Qin F., Huang Z., Zhuang Y., Ma J., Xu H., Shen W. (2016). Hierarchical ZSM‑5 zeolite synthesized by an ultrasound-assisted method as a long-life catalyst for dehydration of glycerol to acrolein. Ind. Eng. Chem. Res..

[71] Kong J., Sheng X., Zhou Y., Zhang Y., Zhou S., Zhang Z. (2014). Ultrasound-assisted synthesis of nanosized hierarchical ZSM-5 and its catalytic performance as the support for heteropolyacid. J. Porous Mater..

[72] Yin X., Liu N., Han M., Xu F., Jia Y., Song F., Cui H. (2023). Ultrasonic-pretreated hydrothermal synthesis of less dense zeolite CHA from the transformation of zeolite T. Ultrason. Sonochem..

[73] Verboekend D., Vile G., Perez-Ramírez J. (2012). Mesopore formation in USY and beta zeolites by base leaching: selection criteria and optimization of pore-directing agents. Cryst. Growth Des..

[74] Li W., Zheng J., Luo Y., Da Z. (2016). Effect of hierarchical porosity and phosphorus modification on the catalytic properties of zeolite Y. Appl. Surf. Sci..

[75] de Jong K.P., Zecevic J., Friedrich H., de Jongh P.E., Bulut M., van Donk S., Kenmogne R., Finiels A., Hulea V., Fajula F. (2010). Zeolite Y crystals with trimodal porosity as ideal hydrocracking catalysts. Angew. Chem. Int. Ed..

[76] Thommes M., Kaneko K., Neimark A.V., Olivier J.P., Rodriguez-Reinoso F., Rouquerol J., Sing K.S.W. (2015). Physisorption of gases, with special reference to the evaluation of surface area and pore size distribution (IUPAC Technical Report). Pure Appl. Chem..

[77] Gil B., Mokrzycki Ł., Sulikowski B., Olejniczak Z., Walas S. (2010). Desilication of ZSM-5 and ZSM-12 zeolites: impact on textural, acidic and catalytic properties. Catal. Today.

[78] Gackowski M., Tarach K., Kuterasiński Ł., Podobiński J., Sulikowski B., Datka J. (2019). Spectroscopic IR and NMR studies of hierarchical zeolites obtained by desilication of zeolite Y: optimization of the desilication route. Microporous Mesoporous Mater..

[79] Ruan Q., Wang T., Miao W., Xiang L., Zhang Q., Le T., Zhang L. (2026). Ultrasonic-assisted synthesis of solid acid catalysts: mechanisms, efficacy, and industrial prospects. Catal. Sci. Technol..

[80] Ahadi N., Askari S., Fouladitajar A., Akbari I. (2022). Facile synthesis of hierarchically structured MIL-53(Al) with superior properties using an environmentally-friendly ultrasonic method for separating lead ions from aqueous solutions. Sci. Rep..

[81] Palčić A., Valtchev V. (2020). Analysis and control of acid sites in zeolites. Appl. Catal. A. Gen..

[82] Murzin D.Y. (2026). Brønsted to Lewis acid site ratio as a kinetic descriptor for catalysis with solid acids. Catal. Today.

[83] Handoko D.S.P. (2023). Triyono, the effect of acid strength of bronsted acid site on the ability of catalyst to break the carbon chain bonds of 1-octadekenes into alkanes and short chain alkenes as a substitute for fossil fuel. Sci. Contrib. Oil Gas.

[84] Suzuki K., Noda T., Katada N., Niwa M. (2007). IRMS-TPD of ammonia: direct and individual measurement of Brønsted acidity in zeolites and its relationship with the catalytic cracking activity. J. Catal..

[85] Yuan Z., Bai Y., Gong K., Huang W. (2024). Accurate measurements of NH_3_ differential adsorption heat unveil structural sensitivity of Brønsted acid and Brønsted/Lewis acid synergy in zeolites. J. Phys. Chem. Lett..

[86] Katada N., Tamura H., Matsuda T., Kawatani Y., Moriwaki Y., Matsuo M., Kato R. (2024). Correlation between Na–Cs Ion exchange properties in the alkaline form and acid strength in the proton form of zeolite. Langmuir.

[87] https://www.sigmaaldrich.com/PL/en/search/tbaoh?focus=products&page=1&perpage=30&sort=relevance&term=TBAOH&type=product (accessed on 23.07.2026).

[88] www.ec.europa.eu/eurostat/statisticsexplained/index.php?title=Electricity_price_statistics (accessed on 23.07.2026).

