# Supplementary material for: On the feasibility of the sonochemical preparation of hierarchical faujasite-type zeolites as an alternative to ultrasonic‑assisted alkaline treatment
Source: Ultrason Sonochem. 2026 Sep 8;133:108046. doi: 10.1016/j.ultsonch.2026.108046 (PMC13586581; doi:10.1016/j.ultsonch.2026.108046)
Supplement: Supplementary file 1 [file mmc1.docx]

**SUPPLEMENTARY DATA**

**Figure S1.** XRD patterns of spent catalysts.

**Table S1.** Differences in costs between the manufacture of desilicated zeolites obtained from post-synthesis alkaline treatment vs. ultrasonically assisted aging of the gel subjected to further hydrothermal treatment. For simplification, the calculations were performed only for the fragments of the syntheses that differed from each other between the two techniques.

| **Post-synthesis alkaline treatment**  **(faujasite) [48]** | **Ultrasonically assisted aging followed by hydrothermal treatment (faujasite) [this work]** |
| --- | --- |
| Commercial zeolite (Zeolyst): 250 € | (Ludox HS 40%): 62 € |
| TBAOH 40 wt. % in H₂O, solution  (Sigma-Aldrich, 250 mL): 225 € | NaOH, pellets (Sigma-Aldrich≥98%, 250 g): 33 € |
| NaOH, pellets (Sigma-Aldrich≥98%, 500 g): 33 € | NaAlO_2_ (Riedel de Haen≥98%, 2.5 kg): 125 € |
| Calcination in a homemade furnace  (5 kW), 14 h: 20 € | Sonicator Q-Sonica400 (60 W), 30 min: 0.01 € |
| Sonicator Q-Sonica400 (60 W), 30 min: 0.01 € | Hydrothermal synthesis  in a heating chamber (3 kW), 24 h: 21 € |
| Total: 528.01 € | Total: 241.01 € |
